# Mastication

**Published:** 1913-01-15

**Authors:** F. C Husband

**Affiliations:** Toronto, Canada


### MASTICATION.

F. C HUSBAND, D.D.S., TORONTO, CANADA.

The oral cavity being the field to which we, as dentists, devote our daily energies, it is reasonable to suppose that we should value its uses more highly than any other branch of the healing art. Research and experimentation by a body of scientific professional men has established the fact that this cavity is the main avenue of bodily health or disease. Its cleanliness is not to satisfy the asthetic, but is a hygienic necessity to reduce micro-organic life and its products to a minimum and to so maintain the vigor of the hard and soft tissues that they may be conditioned to withstand the ever-ready attacks of disease.

The mouth, then, is one of the most important orifices of the body. Being controlled almost entirely by the will, it has become more neglected, ill-kept and improperly used than any other. Only too often does the careless subject wake up to the fact that the masticatory apparatus, intended for a lifetime of usefulness, is fast disappearing. Nature has given us a denture of exceedingly hard texture, and the logical conclusion would seem to be that it was intended for hard work, and that neglect, both as to function and cleanliness, would result in its degeneration. Such indeed is the case. The normal function of the masticatory apparatus has been so ignored and forgotten that a problem of conservation, quite alarming, now confronts us. Cease to use an organ and that organ atrophies. Cease to use the teeth and they become irregular and fall victims to caries and pyorrhea. That is their way of atrophying.

The conservation movement that has been started to deal with these ravages of decay and dental deformities will hope to instill into the minds of the public the necessity of not only observing cleanliness of the masticatory organs, but the proper and thorough use of these organs.

Of what avail is legislation to obtain pure food products and pure water if these things are immediately contaminated the moment they enter an unclean mouth?

All the benefits of cleanliness in prepartion are immediately undone the moment this food enters the unclean mouth, and this condition, coupled with faulty and insufficient mastication, interferes seriously with metabolic results.

In primitive times, when foods were coarser and required the vigorous use of the jaws and teeth, there was little need of such hygienic movements as we have to-day, little need for dentists, and the bodies of these primitive people were stronger and disease less prevalent.

The present methods of preparing foodstuffs that appeal to the eye and palate and that require a minimum of time to consume have a strong tendency to allow the teeth to fall into disuse to a great extent. Little thought is given to the fact that only that portion which is digested is the tissue builder. Little thought is given to the fact that quality counts rather than quantity—that food improperly masticated, *i. e.*, thoroughly comminuted and thoroughly mixed with saliva and acted upon by its digestive ptyalin, will never be thoroughly digested, but will act to a greater or less degree as an irritant to the digestive tract.

In 1860 Mr. J. R. Mummery of Great Britain carried on a series of examinations of the skulls of many ancient and primitive races for evidences of caries. Some of these various tribes lived almost entirely on meat, others subsisted on a mixed diet of vegetable products and meat, and still others lived mainly upon vegetables. In all races examined some evidences of caries were found, but the total percentage, compared with that of to-day, was very low, being less than 25 per cent.

Mr. Mummery also observes that those people living wholly or in part on carbohydrate food were more disposed to dental caries than those relying almost entirely upon meat for food, and that in the advance of civilization the tendencies towards decay of the teeth become greater.

In the present age we are subjected to influences that our forefathers never dreamed of. In our mad rush for mental development nature's laws are transgressed, and the fact is largely overlooked that to have a really healthy, vigorous mind one must have a healthy, vigorous body, A diseased and deformed and ill-fed body cannot possibly possess a powerful mind.

Of the evils most harmful to longevity and posterity, the most insidious is the modern method of preparation of our food material for use. Their softened substances do not force us to masticate, and our habit of getting the most with the least amount of effort allows us to swallow the bolus before the necessary saliva has been mixed therewith. The saliva does not flow as it should because the necessary mechanical forces are not brought to bear on the glands, which forces are the vigorous action of the jaws, tongue, cheeks, etc.

This habit of bolting is a pleasant one since the nature of the preparation of the food gives us an appetizing morsel. So it is that the breaking of the habit will be found difficult.

The adult who has from childhood thoroughly masticated his food will be found, usually, to have all parts of the oral cavity and adjacent parts well developed, with regular nasal passages spacious, mucous membranes strong and healthy.

The habit of masticating cannot be too early taught the child. The physiological worth to the development of the hard and soft tissues within the oral cavity, the essential stimulation to the developing facial bones, the glands and lining membranes in and adjacent thereto, and its indispensable value to further digestion are facts that demand our earnest attention.

Carbohydrates are the chief foods made soft by cooking, and while in this condition they are more readily acted upon by the saliva and its digestive ptyalin. Still they are readily bolted and there is but little excitement to cause a rich flow of saliva. So in the vast majority of cases these carbohydrates pass on unacted upon, except to a slight degree, by the ptyalin, which is the only enzyme that has any digestive action upon carbohydrates.

When saliva is demanded in quantity it becomes more strongly alkaline, and thus not only aids digestion, but prevents bacterial fermentation of deposits remaining after deglutition.

Great exertion of the muscles of mastication excites the flow of lymph, the requisite for salivary formation, and the hardness of the material crushed within the oral cavity, together with the muscular force required, stimulates the flow of blood to the gums and alveolus, where its abundance is so necessary in the rejuvenation of the tissues.

The vigorous use of the teeth and muscles of mastication by the child produces marked development in the facial outline, the jaws, and the roots of the teeth, as well as producing a thick, tough alveolus. Where this development does not take place due to neglect, we usually find irregular teeth, constricted palatine arches, ill-developed nares, no straight septum with generous passages and regular, well-defined turbinates.

When these conditions obtain pathological conditions of the mucous linings throughout the respiratory tract supervene, the narrow nasal passages are inadequately drained, causing inflammation. During the ages from four to sixteen the glandular tissues of the posterior part of the naso-pharynx become hypertrophied, forming adenois. This condition interferes with the free passage of air to the lungs, and mouth breathing results. With the mouth habitually open the lateral pressure of the tongue is lessened, while the buccinator muscle is drawn more tense, and the result is a narrowing of the jaws. Further, the air is not conditioned to enter the lungs, there is dryness of the mouth and throat, augmenting the action of caries, the mucous membrane is kept irritated, and there is developed a good field for the tubercle bacillus.

The development and maintenance of the salivary glands is the result of great muscular activity. The health and strength of their secretions depend upon the flow of blood and lymph in sufficient quantity. During inactivity the flow of lymph almost ceases.

Thought or sight of a tempting morsel causes a flow of saliva and gastric juice. This sense is called appetite, and is the voicing of the body's requirements.

Fletcher has demonstrated the wisdom of keeping the mind on the taste of the food and masticating until we involuntarily swallow such foods as the appetite craves. There will then be no over-indulgence, but the desire for food will depart just as soon as the requirements of the body are met and not later. There can be no indigestion. No media is present in the intestines to develop bacteria, and pathogenic organisms that have escaped the saliva and gastric juice are rendered inactive or die for want of sustenance.

In the long course that food must take in the effort towards tissue building the parts under voluntary control are the terminals only. When the food has passed through and beyond the first three inches of the digestive tract all voluntary treatment ceases.

To the growing youth of to-day is anything dealing with the body's development of more importance than thorough mastication?—*Oral Health.*

